# Determination and Modulation of Total and Surface Calcium-Sensing Receptor Expression in Monocytes In Vivo and In Vitro

**DOI:** 10.1371/journal.pone.0074800

**Published:** 2013-10-01

**Authors:** Julien Paccou, Cédric Boudot, Aurélien Mary, Said Kamel, Tilman Bernhard Drüeke, Patrice Fardellone, Ziad Massy, Michel Brazier, Romuald Mentaverri

**Affiliations:** 1 Department of Rheumatology, University Hospital of Amiens, Amiens, France; 2 INSERM U1088, UFR Médecine/Pharmacie, Université de Picardie Jules Verne, Amiens, France; 3 Department(s) of Clinical Nephrology, University Hospital of Ambroise Paré, Boulogne Billancourt, France; University Heart Center Freiburg, Germany

## Abstract

Expression of the calcium-sensing receptor (CaSR) has previously been demonstrated in human circulating monocytes (HCM). The present study was designed to measure CaSR expression in HCM and to examine its potential modulation by pro-inflammatory cytokines, Ca2+, vitamin D sterols in U937 cell line. Twenty healthy volunteers underwent blood sampling with subsequent isolation of peripheral blood mononuclear cells (PBMC) at 3 visits. Flow cytometry analysis (FACS) was performed initially (V1) and 19 days later (V2) to examine intra- and intersubject fluctuations of total and surface CaSR expression in HCM and 15 weeks later (V3) to study the effect of vitamin D supplementation. *In vitro* experiments were conducted to assess the effects of pro-inflammatory cytokines, calcidiol, calcitriol and Ca2+ on CaSR expression in U937 cell line. By FACS analysis, more than 95% of HCM exhibited cell surface CaSR staining. In contrast, CaSR staining failed to detect surface CaSR expression in other PBMC. After cell permeabilization, total CaSR expression was observed in more than 95% of all types of PBMC. Both total and surface CaSR expression in HCM showed a high degree of intra-assay reproducibility (<3%) and a moderate intersubject fluctuation. In response to vitamin D supplementation, there was no significant change for both total and surface CaSR expression. In the *in vitro* study, U937 cells showed strong total and surface CaSR expression, and both were moderately increased in response to calcitriol exposure. Neither total nor surface CaSR expression was modified by increasing Ca2+ concentrations. Total CaSR expression was concentration dependently decreased by TNFα exposure. In conclusion, CaSR expression can be easily measured by flow cytometry in human circulating monocytes. In the *in vitro* study, total and surface CaSR expression in the U937 cell line were increased by calcitriol but total CaSR expression was decreased by TNFα stimulation.

## Introduction

The tissue calcium concentration is substantially higher in sites of inflammation than in serum. Inflammation may form calcium gradients which in turn modulate the immune response, acting via the calcium-sensing receptor (CaSR) [1]. Originally cloned from bovine parathyroid glands, the CaSR has been studied for its role in mediating systemic calcium homeostasis. However, the CaSR has also been shown to have pleiotropic actions on cells, including modification of cellular proliferation, differentiation, and apoptosis [2]–[4]. CaSR expression has been demonstrated in human circulating monocytes and extracellular calcium has been shown to be a chemokinetic agent for human circulating monocytes [5]–[7]. However, the physiologic role of CaSR expression in human circulating monocytes remains to be elucidated. Noteworthy, vitamin D could be an important regulator of CaSR expression in human circulating monocytes, as functional vitamin D response elements (VDREs) have been identified in both promoters (P1 and P2) of the human CaSR gene and provide the mechanism by which 1,25(OH)2D upregulates CaSR expression in parathyroid chief cells, thyroid C-cells, and renal tubule cells [8]. Pro-inflammatory cytokines such as Interleukin 6 (IL6) are also known to upregulates CaSR gene expression in parathyroid chief cells, thyroid C-cells, and renal tubule cells [9], suggesting that inflammatory diseases may interfere with CaSR expression. We postulated that CaSR expression in circulating monocytes could be particularly useful to follow the impact of vitamin D deficiency and/or pro-inflammatory cytokines. The present study was therefore designed to explore **(i)** whether an easy distinction of CaSR expression in normal human circulating monocytes could be made between that found in the entire cell and that found on cell surface alone, **(ii)** whether CaSR expression could be changed by correction of vitamin D deficiency, and **(iii)** whether *in vitro* exposure to vitamin D sterols and/or Ca2+ and/or pro-inflammatory cytokines could modify CaSR expression using the monocyte U937 cell line.

## Materials and Methods

### Subjects

This study was conducted in 20 healthy volunteer blood donors enrolled from a pool of volunteers set up by the General Clinical Center of University hospital of Amiens, France. All subjects gave their full written informed consent, and the study was approved by the University Hospital ethics committee (Comité de Protection des Personnes Nord-Ouest 2 ou CCP Nord-Ouest 2) (n°2011-A01449-32) and l’Agence Française de Sécurité Sanitaire des Produits de Santé (AFSSAPS). Volunteers were examined by the same staff throughout the study. A questionnaire was used to collect data on each volunteer’s medical history, smoking and drinking habits, and medication (previous and present). Body height and weight were measured using a stadiometer and a balance scale. BMI was calculated as weight/height2 (kg/m2).

### Inclusion and Exclusion Criteria

Inclusion criteria included age over 18 and less than 75 years old. Four groups of 5 subjects were constituted: 18 to 30 years old; 31 to 45 years old; 46 to 60 years old; 61 to 75 years old. Exclusion criteria included the presence of chronic inflammatory disease, ongoing infectious diseases, previous and ongoing cancer, primary hyperparathyroidism, chronic kidney disease and any acute cardiovascular event preceding inclusion. None of the volunteers were cigarette smokers or alcohol drinkers and they did not report any eating disorders, prolonged immobilization or steroid therapy.

### Study Protocol for Volunteers

**Figure 1:**
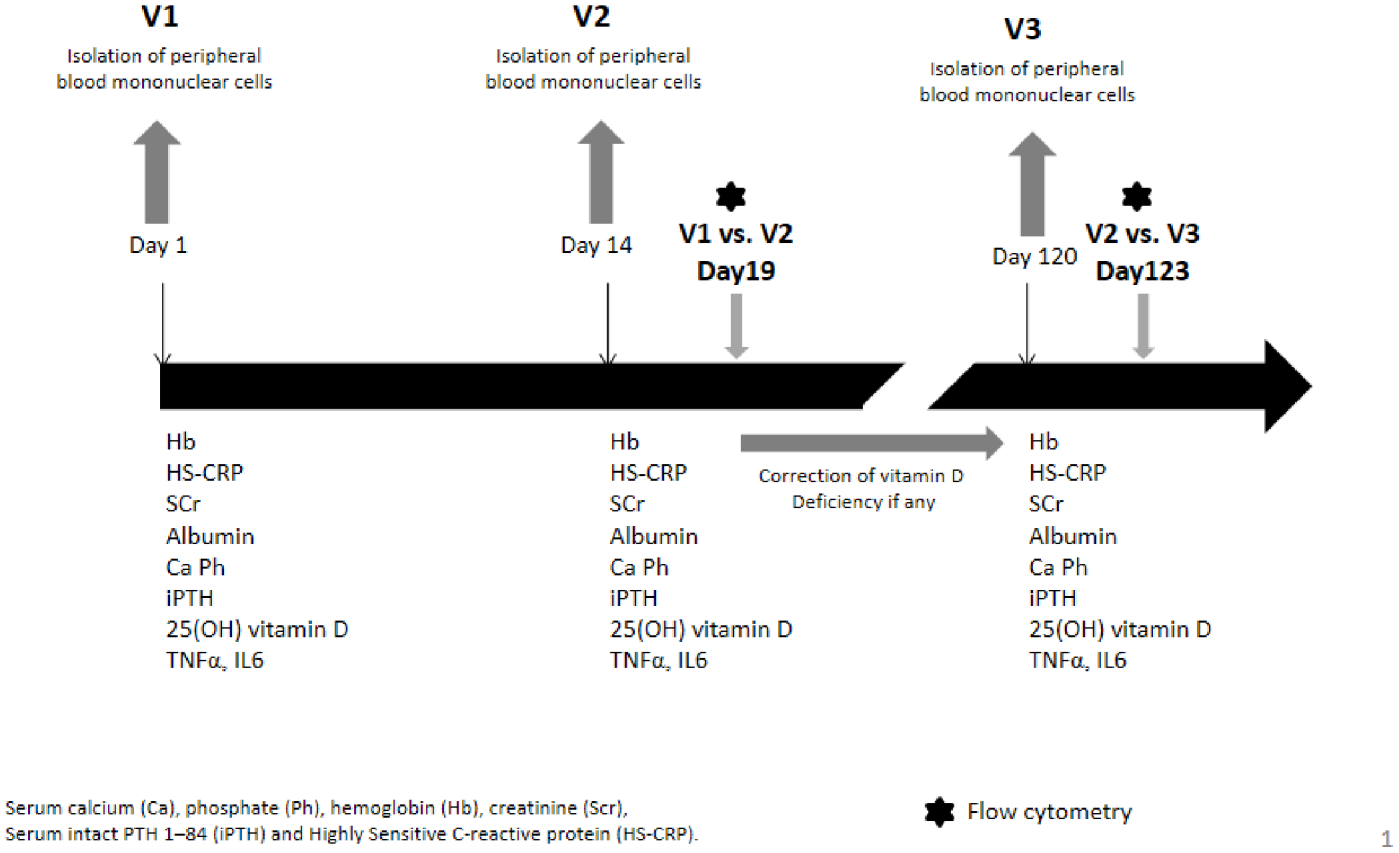
Study protocol.

As shown in Figure 1, morning blood samples were collected at 3 different visits: initially (visit 1 = V1), 19 days later (visit 2 = V2) and again 15 weeks later (visit 3 = V3). As vitamin D deficiency is common in the general population, we hypothesized that it could affect CaSR expression in circulating monocytes. Visit 3 was therefore held to determine CaSR expression after correction of vitamin D deficiency [10]. Vitamin D status was considered to be sufficient when serum 25 OH vitamin D values were ≥30 ng/ml (75 nmol/l). In subjects with 25 OH vitamin D values <30 ng/ml, vitamin D supplementation with vitamin D3 (cholecalciferol, each ampoule containing 100,000 IU) was initiated after V2 and 3 situations have been distinguished: **(i)** when 25 OH vitamin D was <10 ng/ml, one ampoule was administered every 2 weeks for 2 months (i.e. a total of 4 ampoules); **(ii)** when 25 OH vitamin D was between 10 and 20 ng/ml, one ampoule was administered every 2 weeks for 6 weeks (i.e. a total of 3 ampoules); **(iii)** when 25 OH vitamin D was ≥20 ng/ml but <30 ng/ml, one ampoule was administered every 2 weeks for 1 month (i.e. a total of 2 ampoules).

Serum calcium (Ca), phosphate (Pi), albumin (Alb), creatinine (Scr) and highly sensitive C-reactive protein (HS-CRP), and blood hemoglobin (Hb) and white blood cell count were analyzed in an on-site biochemistry laboratory using standard autoanalyzer techniques. Serum intact PTH (iPTH 1–84) was determined by a chemiluminometric immunoassay (Liaison N-tact PTH CLIA; Diasorin, Stillwater, Minnesota, USA). Serum 25 OH vitamin D was assayed using chemiluminescent immunoassay (LIAISON® 25 OH Vitamin D Total Assay, Diasorin, Stillwater, USA). Corrected serum calcium levels were calculated for each healthy volunteer, as measured total serum calcium (mmol/l) +0.02 (40-serum albumin [g/l]), where 40 represents the average serum albumin level in g/l. Estimated glomerular filtration rate (GFR) was calculated by using the Modification of Diet in Renal Disease (MDRD) formula (ml/min). Serum IL6 and TNFα were assayed by ELISA at V1, V2 and V3 (eBiosciences® hIL6 total ELISA BMS213/2 kit and hTNFα total ELISA BMS2034 kit, Vienna, Austria). For hIL6, serum levels may vary from non-detectable to 12.7 pg/ml in healthy subjects (males and females). As to hTNFα, serum levels were not detectable in healthy subjects.

### Isolation of Peripheral Blood Mononuclear Cells

Peripheral blood mononuclear cells were isolated in the afternoon at V1, V2 and V3 in order to perform simultaneous flow cytometry analysis (i) for V1 vs. V2, to examine intra- and intersubject fluctuations of total and surface CaSR expression in human circulating monocytes and (ii) for V2 vs. V3, to study the effect of oral vitamin D supplementation on CaSR expression in human circulating monocytes. After visits V1, V2 and V3, 3 ml of blood from each volunteer was mixed with 22 ml of PBS-0.5%BSA (mononuclear cells were isolated by density gradient centrifugation); 25 ml of diluted blood was carefully added to 10 ml of Lymphosep, and the tubes were then centrifuged for 25 minutes at room temperature at 1800 g. After centrifugation, the interphase containing mononuclear cells was carefully aspirated and the cells were washed using 5 ml of PBS-0.5%BSA, prior to being centrifuged for 5 minutes at room temperature (1800 g). Cells were finally mixed with 1.8 ml of fetal bovine serum (FBS) and 0.2 ml of dimethyl sulfoxide (DMSO) before being divided into four 0.5 ml tubes, which were then frozen at −80°C.

### Flow Cytometry Analysis

After washes in PBS-0.5%BSA, cells were incubated with anti-Calcium Sensing Receptor Monoclonal Antibody (5C10, ADD, Thermo scientific) or negative Control Mouse IgG2a (X 0943, DakoCytomation) for 30 min on ice in the dark. To confirm the specificity of the anti-Calcium Sensing Receptor Monoclonal Antibody, an immunoblot was performed with the same antibody in MDA-MB-231 breast cancer cells and on PBMC, in which the immunizing peptide was used to confirm the specificity of binding (See Figure S1 in supporting information). After washes in PBS-0.5%BSA, cells were incubated with PE-conjugated IgG mouse antibodies (Polyclonal Goat Anti-Mouse Immunoglobulins/RPE [R 0480, DakoCytomation]) for 30 min on ice in the dark. After washes in PBS-0.5%BSA, cells were incubated with monoclonal anti-CD14 antibodies human conjugated to FITC (130-080-701, MACS Miltenyi Biotec) or with monoclonal Mouse IgG2a isotype control antibodies conjugated to FITC (130-091-837, MACS Miltenyi Biotec) for 30 min on ice in the dark. After several washes, surface and total CaSR expression were analyzed by FACS (Aria cytometer). To assay total CaSR expression, detection of intracellular antigens requires a cell permeabilization step prior to immunostaining. For this purpose, cells were incubated with 100 µl of BD Cytofix/Cytoperm™ (Cat. No. 554722) solution for 20 min on ice in the dark and then washed twice with BD Perm/Wash buffer (Cat. No. 554723), prior to being exposed to both primary and secondary antibodies for assessment of total CaSR expression.

### Cell Culture and Stimulation

U937 cell line was purchased from American Type Culture Collection (ATCC, Manassas, USA). Roswell Park Memorial Institute Medium (RPMI), phosphate buffered saline (PBS), trypsin-EDTA and penicillin/streptomycin were provided by Sigma-Aldrich, Saint-Quentin Fallavier, France. GlutaMAX™ was provided by Invitrogen. Recombinant human IL-6 and recombinant human TNFα were provided by R&D systems®, Chartres-de-Bretagne, France. Calcidiol and calcitriol were provided by Sigma-Aldrich, Saint-Quentin Fallavier, France. Human U937 monocytes/macrophage cells were maintained in RPMI supplemented with 10% fetal bovine serum (FBS), 2.0 mM GlutaMAX™, 100 U/ml penicillin/streptomycin, at 37°C and 5% CO2. Cells were cultured up to passage 20, and then discarded. Before each experiment, cells were serum starved and preincubated for 24 hours with TNFα (0, 10, 50 and 100 ng/ml), IL6 (0, 10, 50 and 100 ng/ml), Ca^2+^ (0.4, 0.8, 1.2 and 1.6 mM), calcidiol (0, 10, 100 and 1000 nM) and calcitriol (0, 0.01, 0.1, 1.0 and 10 nM). Stimulation was stopped by aspirating the medium and washing the cells with ice-cold PBS-0.5%BSA. Surface and total CaSR expression were analyzed by FACS (FACS Aria cytometer).

### Statistical Methods

To describe the characteristics of the population, quantitative variables were expressed as mean ± standard deviation (SD), median and range and qualitative variables were expressed as percentage with a 95*%* confidence interval. Spearman correlation coefficients were used to measure a statistical dependence between two quantitative variables. Mann-Whitney test was used for comparison of quantitative variables between two independent groups, while Chi-square or Fisher’s test was used for comparison of categorical data. Wilcoxon test was used for comparison of quantitative variables between two related samples. Friedman test was used to compare more than two repeated observations on the same subjects. When the test was significant, *post hoc* comparisons were performed with Wilcoxon test and p values were adjusted by the Holm method. Similarly, Kruskal-Wallis test was used to compare more than two independent samples. When the test was significant, *post hoc* comparisons were performed with Mann-Whitney test and p values were adjusted with the Holm method. Univariate and multivariate linear regression models were used to analyze factors associated with total and surface CaSR expression. Data obtained simultaneously for V1 and V2 (V1+V2) and for V2 and V3 (V2+V3) were pooled. The significance level was 5% for all analyses. The significance level was 20% for univariate linear regression models, and 5% for multivariate linear regression models. SAS software version 9.2 (SAS Institute, Cary, NC) and R 2.14.1 software were used for all analyses.

## Results

### Demographic Data

The mean age of the subjects was 44.2 years (22 to 73 years), 35% were male and the mean body mass index was 25.2 kg/m2 (±4.4). Four subjects presented hypercholesterolemia (20%) and 3 of them were hypertensive. Two of them also presented diabetes mellitus. Two patients were taking a vitamin D3 supplement before inclusion in the study.

### Serum Biochemistry

As shown in Table 1, biochemical values were compared between V1, V2 and V3. Serum albumin (p = 0.0003), hemoglobin (p = 0.02) and serum intact PTH (p = 0.04) were significantly different between V1, V2 and V3. Serum calcium was also significantly different between V1, V2 and V3 (p = 0.003), but corrected serum calcium values were not significantly different (p = 0.58). As expected, 25 OH vitamin D levels after supplementation were also different between V1, V2 and V3 (p = 0.0003). No statistical differences were observed for TNFα (p = 0.74) and IL6 (p = 0.07).

**Table 1:** Biochemical characteristics in volunteer blood donors.

	V1	V2	V3	P
Serum Ca (mmol/l)	2.28±0.09	2.23±0.08	2.27±0.08	**0.003** V1 vs V2 p = 0.006 V2 vs V3 p = 0.03
Serum PO_4_ (mmol/l)	0.94±0.18	0.94±0.2	1.01±0.16	0.22
Serum Albumin (g/l)	43.8±2.79	41.1±4.29	43.7±3.7	**0.0003** V1 vs V2 p = 0.006 V2 vs V3 p = 0.0008
Corrected serum calcium (mmol/l)	2.19±0.07	2.20±0.08	2.18±0.08	0.58
25-(OH)-Vit D (ng/ml)	18.66±10.78	18.86±12.4	39.6±8.3	**0.0003** V1 vs V3 p = 0.0003 V2 vs V3 p = 0.0008
Serum PTH (pg/ml)	36.9±24.44	40.95±24.2	35.3±23.7	**0.04**
Serum creatinine (µmol/l)	87.17±13.97	90.0±12.15	88.8±13.4	0.29
Creatinine clearance MDRD (ml/min)	75±11.22	72±11.10	72±12.05	0.13
Hemoglobin (g/dl)	14.8±1.29	14.6±1.41	14.2±1.07	**0.02** V1 vs V3 p = 0.04
Neutrophils (/µl)	3590±1050	3215±1236	3183±1021	0.22
Monocytes (/µl)	410±129	435±172	455±138	0.30
HSCRP (mg/l)	2.24±3.3	2.76±4.3	2.4±3.4	0.51
IL6 (pg/ml)	0.39±0.19	0.29±0.18	0.25±0.2	0.07
TNFα (pg/ml)	51.3±44.6	40.2±42.0	38.1±30.9	0.74

Data are expressed as mean ± SD. Ca, serum calcium; HSCRP, Highly sensitive C-reactive protein; PO_4_, serum phosphate; PTH, parathyroid hormone; MDRD, modification of Diet in Renal Disease (MDRD) formula (ml/min); IL6, Interleukin-6; TNFα, Tumor Necrosis Factor-α.

### Comparison of CaSR Expression in Human Circulating Monocytes: V1 vs. V2 and V2 vs. V3

By FACS analysis, more than 95% of human circulating monocytes exhibited positive cell surface CaSR staining. In contrast, CaSR staining failed to detect surface CaSR expression in other PBMC. After cell permeabilization, total CaSR expression was detected in more than 95% of all types of PBMC (see Figures S2 and S3 in supporting information). Both total and surface CaSR expression in HCM showed (i) a high degree of intra-assay reproducibility (<3%) and an approximately 10% variation from V1 to V2 (ii) a moderate intersubject fluctuation observed for each FACs analysis at V1, V2 or V3 (Table 2). Oral Vitamin D supplementation did not induce a change in either total or surface CaSR expression of HCM (V3 vs. V2 determinations) (Table 2).

**Table 2:** Simultaneous flow cytometry analysis of surface and total calcium sensing receptor (CaSR) expression in circulating monocytes for V1 vs. V2 and V2 vs. V3.

	V1	V2	P-value
Surface CaSR expression	**1221±359** [1053–1389]	**1187±401** [999–1375]	**N.S.**
	**V1**	**V2**	
Total CaSR expression	**544±155** [469–619]	**467±105** [418–516]	**N.S.**
	**V2**	**V3**	
Surface CaSR expression	**1218±370** [1040–1397]	**1258±437** [1033–1482]	**N.S.**
	**V2**	**V3**	
Total CaSR expression	**971±206** [865–1077]	**1199±446** [969–1428]	**N.S.**

N.S., non-significant.

### Linear Regression Analysis and Correlations

V1+V2: Univariate linear regression analysis of surface CaSR expression demonstrated a significant correlation only with serum phosphate (p = 0.048). A significant correlation was demonstrated between total CaSR expression and corrected serum calcium (p = 0.032). According to international accepted rules, a borderline significance was found for hemoglobin (p = 0.099), 25 OH vitamin D (p = 0.075) and GFR estimated by MDRD (p = 0.094). Multivariate linear regression analysis revealed a significant correlation between total CaSR expression and corrected serum calcium (p = 0.014, r = 0.48) (see Tables S1 and S2 in supporting information). V2+V3: Univariate linear regression analysis failed to demonstrate any significant correlations for surface and total CaSR cell expression.

### *In vitro* Experiments in U937 Cells

**Figure 2:**
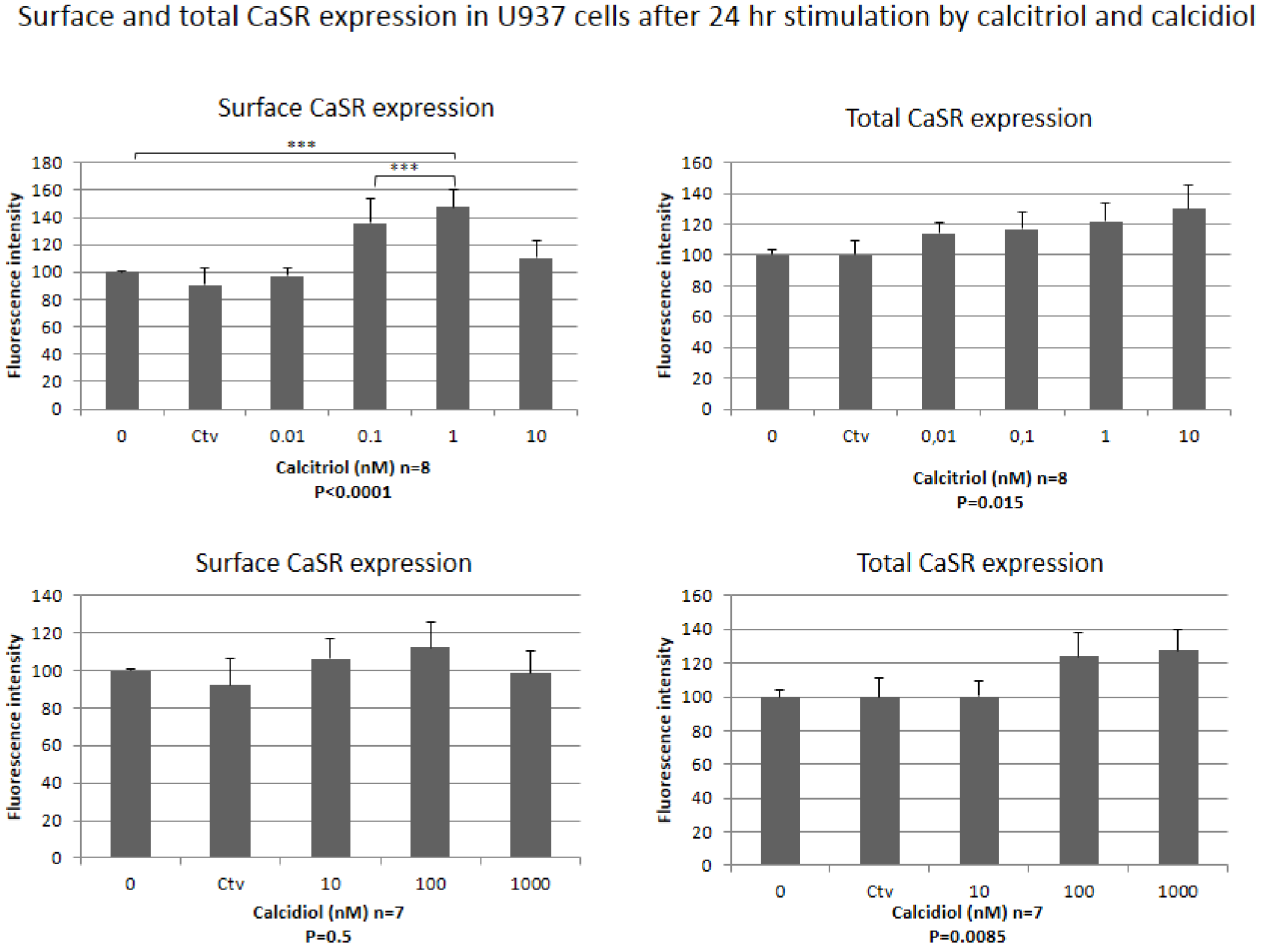
Surface and total CaSR expression in U937 cells after 24-hour exposure to increasing concentrations of calcitriol and calcidiol in incubation medium, respectively.

**Figure 3:**
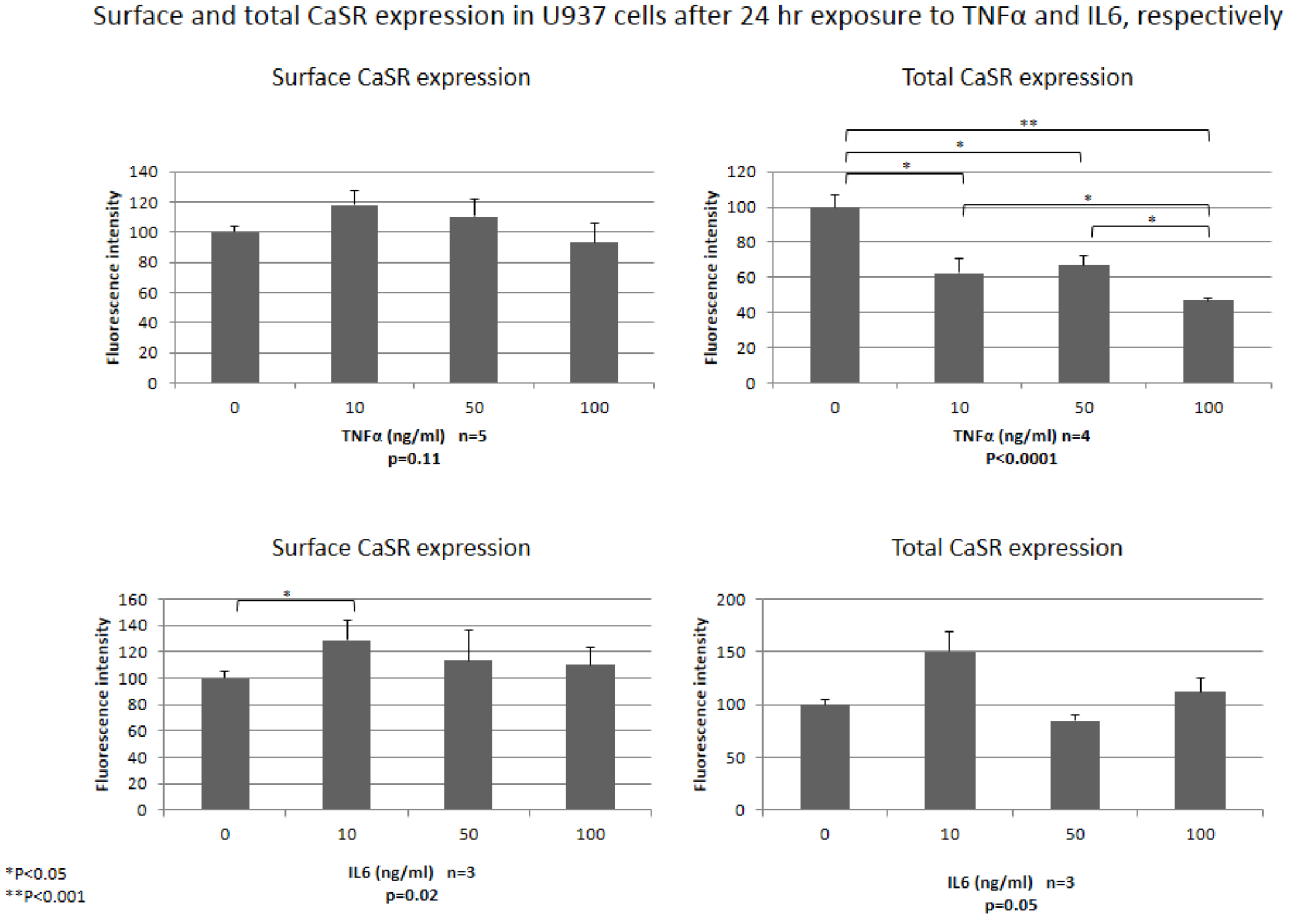
Surface and total CaSR expression in U937 cells after 24-hour exposure to increasing concentrations of TNFα and IL6 in incubation medium, respectively.

We further examined the *in vitro* effects of Ca2+, calcidiol and calcitriol on CaSR expression in human U937 cells. Like human circulating monocytes, U937 cells also showed strong total and surface CaSR cell expression, present in more than 95% of cells. After incubation of U937 cells for 24 hr in presence of increasing concentrations of Ca2+, ranging from 0.4 to 1.6 mM, no significant differences were observed for either total or surface CaSR expression (p = 0.18 and p = 0.78, respectively) (see Figure S4 in supporting information). Calcitriol, added to cultures for 24 hr at concentrations ranging from 0.01 nM to 10 nM, significantly stimulated both total and surface CaSR expression (p = 0.015 and p<0.0001, respectively). The action of calcitriol on total CaSR expression showed a concentration-dependent trend (Figure 2). Surface CaSR expression was significantly increased by calcitriol with a peak increase of 47.7% observed when cells were cultured for 24 h in presence of 1.0 nM calcitriol (p<0.01). Calcidiol had a significant overall effect only on total CaSR expression of U937 cells (p = 0.008). Finally, we examined CaSR expression in human U937 cells in response to TNFα and IL6 stimulation. Surface and total CaSR expression was analyzed by flow cytometry after incubation of cells for 24 hr with various concentrations of TNFα and IL6 (Figure 3). Although TNFα had no effect on surface CaSR expression, total CaSR expression was dose-dependently decreased by exposure to this cytokine (p = 0.009), with a peak effect observed at 100 ng/ml (−53.7%). For IL6 stimulation, no clear-cut trend was observed for both total and surface CaSR expression.

## Discussion

By FACS analysis, more than 95% of human circulating monocytes exhibited positive cell surface CaSR staining whereas surface CaSR staining was negative in all other types of PBMC. However, after cell permeabilization total CaSR expression was observed in more than 95% of all types of PBMC. Total and surface CaSR expression in circulating monocytes from volunteer blood donors, as measured by flow cytometry, showed a high degree of intra-assay reproducibility and a moderate intersubject fluctuation. Total or surface CaSR expression of human circulating monocytes was not modified by oral vitamin D supplementation and did not appear to be related to any of the biochemical parameters measured except for an association found between corrected serum calcium and total CaSR expression. In the *in vitro* experiments, U937 cells also exhibited strong surface and total CaSR expression. Exposure of U937 cells to calcitriol stimulated both surface and total CaSR expression, with similar, but more modest effects of calcidiol on total CaSR expression. Finally total, but not surface, CaSR expression was dramatically and dose-dependently decreased by exposure to TNFα. For IL6 stimulation, no clear-cut trend was observed for both total and surface CaSR expression. Neither total nor surface CaSR expression was modified by exposure to moderately decreased or increased Ca2+ concentrations in the incubation milieu.

The calcium-sensing receptor (CaSR) is a G protein–coupled cell surface receptor that is able to “sense” extracellular calcium ions. It enables the cell to respond to small changes in extracellular ionized calcium concentration [11]. Although the CaSR is mainly expressed in the parathyroid gland, kidney and bone [12]–[15] it is present in many other tissues. Preliminary data suggest that the CaSR is also expressed in the mouse J774 cell line (macrophage/monocyte cell line) and senses local changes of Ca2+, thereby inducing chemotaxis and cell proliferation [15]. Olszak et al. have provided evidence that the CaSR is essential for the chemotactic response of monocytes to Ca2+ by showing that monocytes derived from CaSR-deficient mice lacked the normal chemotactic response to a calcium gradient [7]. Yamaguchi et al. reported that human peripheral blood monocytes express CaSR protein, as assessed by immunocytochemistry, flow cytometry and Western blot analysis, as well as CaSR transcript as assessed by RT-PCR [16]. To our knowledge, we show for the first time total CaSR expression in circulating monocytes by flow cytometry analysis. Of all the cell types present in circulating blood, monocytes are particularly good candidates for sensing changes in serum Ca2+. Here we show that indeed total CaSR expression in circulating monocytes from healthy volunteers was associated with the concentration of corrected serum calcium. However, we failed to observe changes in surface or total CaSR expression of U937 cells in vitro in response to changes in Ca2+ concentration in the incubation medium. As the Ca2+ concentration was limited to 0.4–1.6 mM in our experiments, we cannot exclude the possibility of *in vivo* agonist-driven insertional signaling (ADIS), which would explain these discordant results. Hence, as demonstrated by Grant et al., in HEK-transfected cells, endogenous agonists of the CaSR, such as Mg2+, spermine and cyclic amino acids, may increase the net plasma membrane abundance of the receptor in vivo, without reaching 5 mM Ca2+ [17]. Noteworthy, monocytes/macrophages are thought to migrate inflammation sites which are often associated with high Ca2+ concentrations. Taken together, our observations suggest that the CaSR expressed by circulating monocytes could potentially represent an easily accessible biomarker influenced by Ca2+ extracellular concentrations. For this purpose, FACs calibration will be necessary before conducting further experiments.

Functional vitamin D response elements (VDREs) have been identified in both promoters (P1 and P2) of the human CaSR gene and provide the mechanism by which 1,25(OH)2D upregulates CaSR expression in parathyroid chief cells, thyroid C cell, and renal tubule cells [8]. CaSR expression in circulating monocytes from our volunteer blood donors was not modified by cholecalciferol supplementation, which corrected calcidiol levels towards the range of normal values. However, the stimulation by supra-physiological or pharmacological doses of calcidiol and calcitriol was able to upregulate CaSR expression in U937 cells. It is important to note that no effect of calcidiol was observed on surface CaSR expression and that only supraphysiological concentrations of calcidiol had an effect on total CaSR expression. Further work is needed to clarify this issue and identify the cellular mechanisms behind it. Such work is ongoing in our laboratory.

Pro-inflammatory cytokines such as IL6 are also known to upregulate CaSR gene expression [9]. In the present study, FACS analysis after IL6 stimulation showed no clear-cut trend in CaSR expression but, surprisingly, its expression was downregulated by TNFα stimulation. This is an interesting finding because monocytes are known to be the main producers of TNFα in innate immune responses and in chronic inflammatory diseases such as rheumatoid arthritis but little is known about the effects of TNFα on monocytes themselves [18].

The present study provides evidence indicating that CaSR expression can be easily measured by flow cytometry in human circulating monocytes. Measurement of CaSR expression would be potentially useful in certain clinical situations, in which changes in CaSR expression could be expected. Further studies are necessary to determine how pathological conditions involving monocytes/macrophages, such as rheumatoid arthritis [19], [20], atherosclerosis [21], [22] or osteoporosis [23], [24], modify CaSR expression.

## Supporting Information

Figure S1 **CaSR expression in MDA-MB-231 breast cancer cells and peripheral blood mononuclear cells (PBMC).** (A) Expression of CaSR and FLAG was confirmed by western blot in MDA-MB-231 transfected with empty vector (E) and wild-type CaSR (WT). (B) Expression of CaSR was confirmed by western blot in peripheral blood mononuclear cells and antibodies specificity anti-CaSR was tested using Blocking peptide method.
(TIF)

Figure S2 **Flow cytometry analysis of Surface CaSR expression in Monocytes CD14+.**
(TIF)

Figure S3 **Flow cytometry analysis of Total CaSR expression in Monocytes CD14+.**
(TIF)

Figure S4 **Surface and total CaSR expression in U937 cells after 24-hr stimulation by Ca2+.**
(TIF)

Table S1 **Linear regression analysis of surface CaSR expression for V1+V2.**
(DOCX)

Table S2 **Linear regression analysis of total CaSR expression for V1+V2.**
(DOCX)

## References

[1] Quinn SJ, Kifor O, Trivedi S, Diaz R, Vassilev P, et al (1998) Sodium and ionic strength sensing by the calcium receptor. J Biol Chem 273: 19579–86. 9677383 10.1074/jbc.273.31.19579

[2] Brown EM, Quinn S, Vassilev PM, Hebert S (1999) G-protein-coupled, extracellular Ca2+-sensing receptor: a versatile regulator of diverse cellular functions. Vitam Horm 55: 1–71. 9949679 10.1016/s0083-6729(08)60933-4

[3] Lin KI, Chattopadhyay N, Bai M, Alvarez R, Dang CV, et al (1998) Elevated extracellular calcium can prevent apoptosis via the calcium-sensing receptor. Biochem Biophys Res Commun 249: 325–331. 9712695 10.1006/bbrc.1998.9124

[4] Freichel M, Zink-Lorenz A, Holloschi A, Hafner M, Flockerzi V, et al (1996) Expression of a calcium-sensing receptor in a human medullary thyroid carcinoma cell line and its contribution to calcitonin secretion. Endocrinology 137: 3842–3848. 8756555 10.1210/endo.137.9.8756555

[5] Yamaguchi T, Olozak I, Chattopadhyay N, Butters RR, Kifor O, et al (1998) Expression of extracellular calcium (Ca2+o)-sensing receptor in human peripheral blood monocytes. Biochem Biophys Res Commun 246: 501–6. 9610391 10.1006/bbrc.1998.8648

[6] House MG, Kohlmeier L, Chattopadhyay N, Kifor O, Yamaguchi T, et al (1997) Expression of an extracellular calcium sensing receptor in human and mouse bone marrow cells. J Bone Miner Res 12: 1959–70. 9421228 10.1359/jbmr.1997.12.12.1959

[7] Olszak IT, Poznansky MC, Evans RH, Olson D, Kos C, et al (2000) Extracellular calcium elicits a chemokinetic response from monocytes in vitro and in vivo. J Clin Invest 105: 1299–1305. 10792005 10.1172/JCI9799 PMC315448

[8] Canaff L, Hendy GN (2002) Human calcium-sensing receptor gene. Vitamin D response elements in promoters P1 and P2 confer transcriptional responsiveness to 1,25-dihydroxyvitamin D. J Biol Chem 33: 30337–50. 10.1074/jbc.M201804200 12036954

[9] Canaff L, Zhou X, Hendy GN (2008) The proinflammatory cytokine, interleukin-6, up-regulates calcium-sensing receptor gene transcription via Stat1/3 and Sp1/3. J Biol Chem 20: 13586–600. 10.1074/jbc.M708087200 18348986

[10] Souberbielle JC, Prié D, Courbebaisse M, Friedlander G, Houillier P, et al (2008) Update on vitamin D and evaluation of vitamin D status. Ann Endocrinol (Paris) 69: 501–10. 18804195 10.1016/j.ando.2008.07.010

[11] Brown EM, MacLeod RJ (2001) Extracellular calcium sensing and extracellular calcium signaling. Physiol Rev 81: 239–97. 11152759 10.1152/physrev.2001.81.1.239

[12] Adams GB, Chabner KT, Alley IR, Olson DP, Szczepiorkowski ZM, et al (2006) Stem cell engraftment at the endosteal niche is specified by the calcium-sensing receptor. Nature (Lond) 439: 599–603. 16382241 10.1038/nature04247

[13] Yamaguchi T, Chattopadhyay N, Kifor O, Butters RR, Sugimoto T, et al (1998) Mouse osteoblastic cell line (MC3T3-E1) expresses extracellular calcium (Ca2+o)-sensing receptor and its agonists stimulate chemotaxis and proliferation of MC3T3-E1 cells. J Bone Miner Res 13: 1530–38. 9783541 10.1359/jbmr.1998.13.10.1530

[14] Kanatani M, Sugimoto T, Kanzawa M, Yano S, Chihara K (1999) High extracellular calcium inhibits osteoclast-like cell formation by directly acting on the calcium-sensing receptor existing in osteoclast precursor cells. Biochem Biophys Res Commun 261: 144–48. 10405337 10.1006/bbrc.1999.0932

[15] Yamaguchi T, Kifor O, Chattopadhyay N, Bai M, Brown EM (1998) Extracellular calcium (Ca2+o)-sensing receptor in a mouse monocyte-macrophage cell line (J774): potential mediator of the actions of Ca2+o on the function of J774 cells. J Bone Miner Res 13: 1390–1397. 9738511 10.1359/jbmr.1998.13.9.1390

[16] Yamaguchi T, Olozak I, Chattopadhyay N, Butters RR, Kifor O, et al (1998) Expression of extracellular calcium (Ca2+o)-sensing receptor in human peripheral blood monocytes. Biochem Biophys Res Commun 246: 501–6. 9610391 10.1006/bbrc.1998.8648

[17] Grant MP, Stepanchick A, Cavanaugh A, Breitwieser GE (2011) Agonist-driven maturation and plasma membrane insertion of calcium-sensing receptors dynamically control signal amplitude. Sci Signal 4: ra78. 22114145 10.1126/scisignal.2002208

[18] Rushworth SA, Shah S, MacEwan DJ (2011) TNF mediates the sustained activation of Nrf2 in human monocytes. J Immunol 187: 702–7. 21670314 10.4049/jimmunol.1004117

[19] Kinne RW, Brauer R, Stuhlmuller B, Palombo-Kinne E, Burmester GR (2000) Macrophages in rheumatoid arthritis. Arthritis Res 2: 189–202. 11094428 10.1186/ar86 PMC130001

[20] Davignon JL, Hayder M, Baron M, Boyer JF, Constantin A, et al (2013) Targeting monocytes/macrophages in the treatment of rheumatoid arthritis. Rheumatology 52: 590–598. 23204551 10.1093/rheumatology/kes304

[21] Tintut Y, Patel J, Territo M, Saini T, Parhami F, et al (2002) Monocyte/macrophage regulation of vascular calcification in vitro. Circulation 105: 650–5. 11827934 10.1161/hc0502.102969

[22] Gratchev A, Sobenin I, Orekhov A, Kzhyshkowka J (2012) Monocytes as a diagnosis marker of cardiovascular disease. Immunobiology 217: 476–482. 22325375 10.1016/j.imbio.2012.01.008

[23] Wang Y, Li L, Moore BT, Peng XH, Fang X, et al (2012) MiR-133a in human circulating monocytes: a potential biomarker associated with postmenopausal osteoporosis. PloS One 7: e34641. 22506038 10.1371/journal.pone.0034641 PMC3323546

[24] Liu YZ, Dvornyk V, Lu Y, Shen H, Lappe JM, et al (2005) A novel pathophysiological mechanism for osteoporosis is suggested by an in vivo gene expression study of circulating monocytes. J Biol Chem 280: 29011–6. 15965235 10.1074/jbc.M501164200

