# Supplementary figures and images for: Determination and Modulation of Total and Surface Calcium-Sensing Receptor Expression in Monocytes In Vivo and In Vitro
Source: PLoS One. 2013 Oct 1;8(10):e74800. doi: 10.1371/journal.pone.0074800 (PMC3788033; doi:10.1371/journal.pone.0074800)

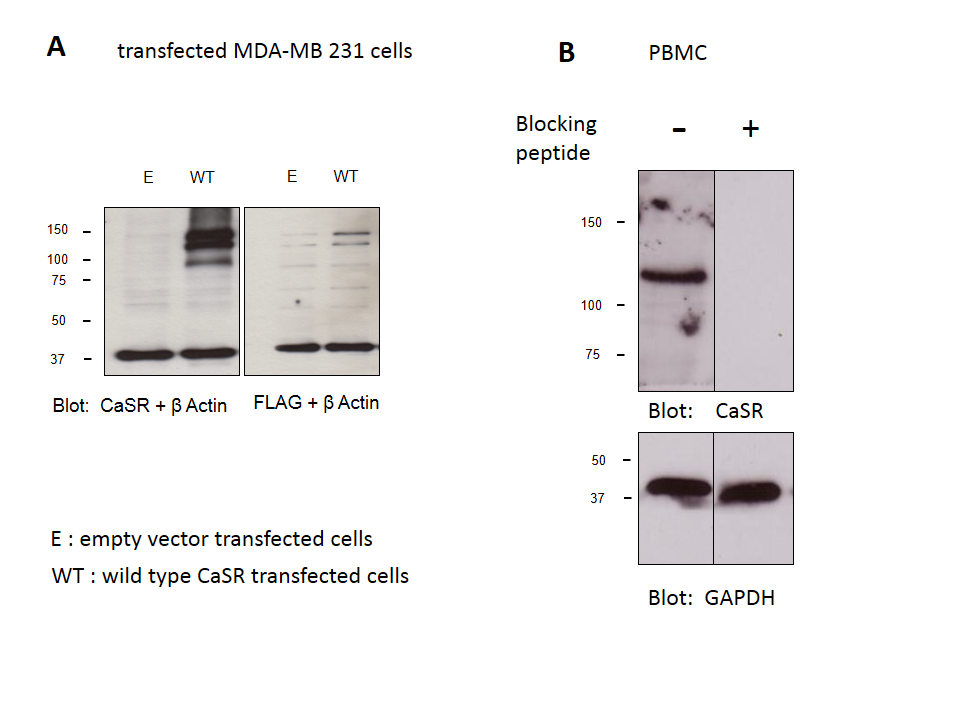

Supplement: Figure S1 — CaSR expression in MDA-MB-231 breast cancer cells and peripheral blood mononuclear cells (PBMC). (A) Expression of CaSR and FLAG was confirmed by western blot in MDA-MB-231 transfected with empty vector (E) and wild-type CaSR (WT). (B) Expression of CaSR was confirmed by western blot in peripheral blood mononuclear cells and antibodies specificity anti-CaSR was tested using Blocking peptide method. (TIF) [file pone.0074800.s001.tif]

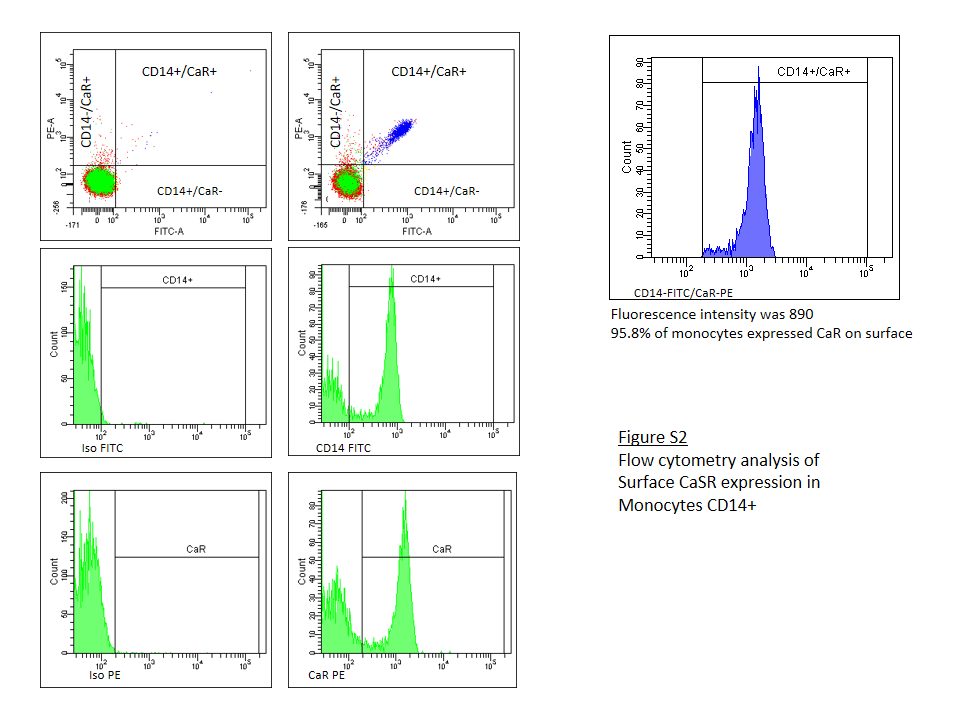

Supplement: Figure S2 — Flow cytometry analysis of Surface CaSR expression in Monocytes CD14+. (TIF) [file pone.0074800.s002.tif]

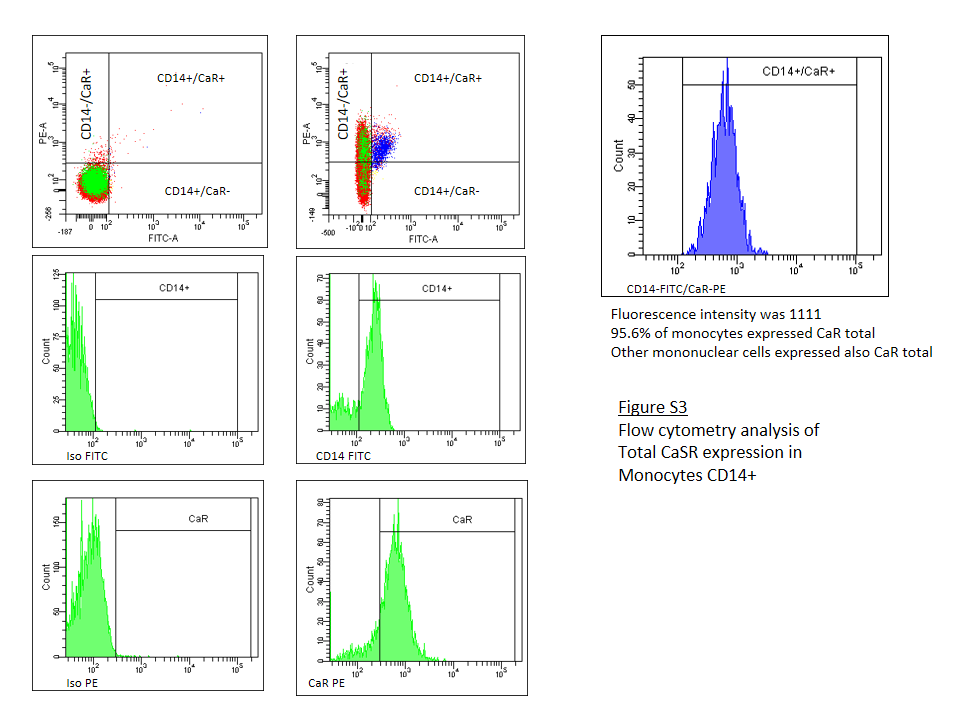

Supplement: Figure S3 — Flow cytometry analysis of Total CaSR expression in Monocytes CD14+. (TIF) [file pone.0074800.s003.tif]

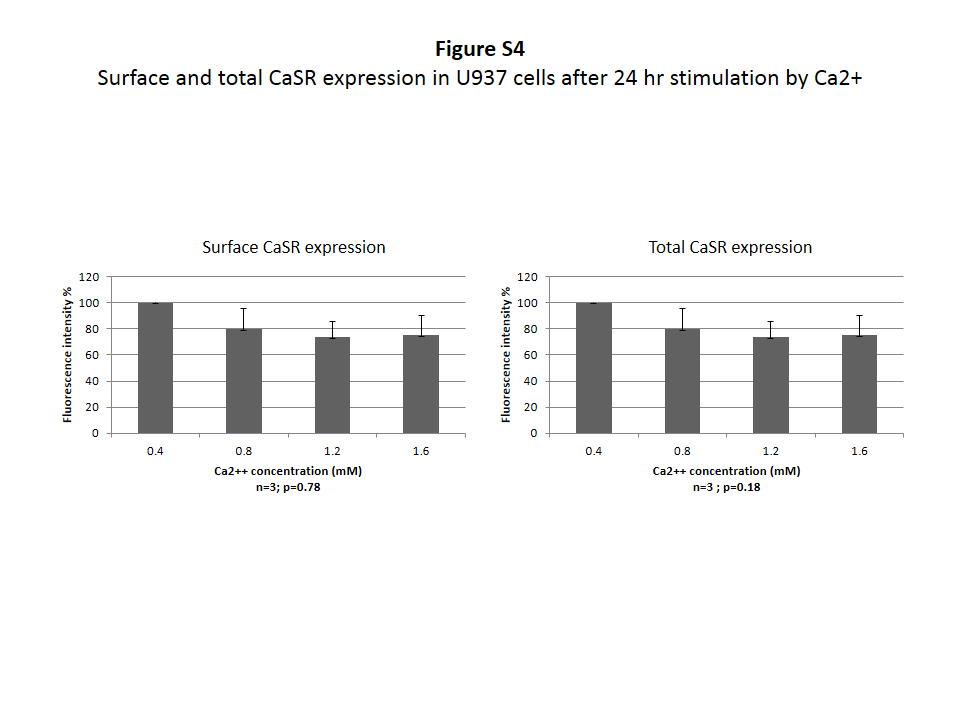

Supplement: Figure S4 — Surface and total CaSR expression in U937 cells after 24-hr stimulation by Ca2+. (TIF) [file pone.0074800.s004.tif]
