# Supplementary material for: Determination and Modulation of Total and Surface Calcium-Sensing Receptor Expression in Monocytes In Vivo and In Vitro
Source: PLoS One. 2013 Oct 1;8(10):e74800. doi: 10.1371/journal.pone.0074800 (PMC3788033; doi:10.1371/journal.pone.0074800)
Supplement: Table S1 — Linear regression analysis of surface CaSR expression for V1+V2. (DOCX) [file pone.0074800.s005.docx]

**Supplementary Material**

**Table 1: Linear regression analysis of surface CaSR expression for V1+V2**

|  | **P-value for univariate model** | **P-value for multivariate model** |
| --- | --- | --- |
| Serum calcium | 0.129 | 0.159 |
| Corrected serum calcium | 0.177 | 0.646 |
| Serum phosphate | **0.048** | 0.048 |
| Serum 25 OH Vitamin D | 0.169 | 0.379 |
| HS-CRP | 0.644 |  |
| Serum albumin | 0.675 |  |
| Hemoglobin | 0.824 |  |
| Monocytes | 0.614 |  |
| Serum intact PTH | 0.728 |  |
| GFR-MDRD | 0.892 |  |
| Serum creatinine | 0.739 |  |
| Age | 0.229 |  |
| Serum TNFα | 0.486 |  |
| Serum IL6 | 0.825 |  |

Hs-CRP, highly sensitive C-reactive protein; GFR-MDRD, estimated glomerular filtration rate calculated using Modification of Diet in Renal Disease formula (ml/mn); TNFα, tumor necrosis factor α; IL6, Interleukin-6.
