# Supplementary material for: Determination and Modulation of Total and Surface Calcium-Sensing Receptor Expression in Monocytes In Vivo and In Vitro
Source: PLoS One. 2013 Oct 1;8(10):e74800. doi: 10.1371/journal.pone.0074800 (PMC3788033; doi:10.1371/journal.pone.0074800)
Supplement: Table S2 — Linear regression analysis of total CaSR expression for V1+V2. (DOCX) [file pone.0074800.s006.docx]

**Supplementary Material**

**Table 2: Linear regression analysis of total CaSR expression for V1+V2**

|  | **P-value for univariate model** | **P-value for multivariate model** |
| --- | --- | --- |
| Serum calcium | 0.417 |  |
| Corrected serum calcium | **0.032** | **0.014** |
| Serum phosphate | 0.645 |  |
| Serum 25 OH Vitamin D | **0.075** | 0.195 |
| HS-CRP | 0.134 | 0.159 |
| Serum albumin | 0.342 |  |
| Hemoglobin | **0.099** | 0.058 |
| Monocytes | 0.215 |  |
| Serum intact PTH | 0.474 |  |
| GFR-MDRD | **0.094** | 0.616 |
| Serum creatinine | 0.593 |  |
| Age | 0.287 |  |
| TNFα | 0.421 |  |
| IL6 | 0.800 |  |

HS-CRP, highly sensitive C-reactive protein; GFR-MDRD, estimated glomerular filtration rate calculated using Modification of Diet in Renal Disease formula (ml/min); TNFα, tumor necrosis factor α; IL6, Interleukin-6; N.S., non significant.
